# Health Benefits of Palm Tocotrienol-Rich Fraction: A Systematic Review of Randomized Controlled Trials

**DOI:** 10.1093/nutrit/nuae061

**Published:** 2024-06-25

**Authors:** Aaron Deming Looi, Uma Devi Palanisamy, Mohanambal Moorthy, Ammu K Radhakrishnan

**Affiliations:** Jeffrey Cheah School of Medicine and Health Sciences, Monash University Malaysia, Jalan Lagoon Selatan, 47500 Sunway, Malaysia; Jeffrey Cheah School of Medicine and Health Sciences, Monash University Malaysia, Jalan Lagoon Selatan, 47500 Sunway, Malaysia; Jeffrey Cheah School of Medicine and Health Sciences, Monash University Malaysia, Jalan Lagoon Selatan, 47500 Sunway, Malaysia; Jeffrey Cheah School of Medicine and Health Sciences, Monash University Malaysia, Jalan Lagoon Selatan, 47500 Sunway, Malaysia

**Keywords:** vitamins, nutrients, inflammation, health, tocotrienol

## Abstract

**Context:**

Vitamin E, a well-known antioxidant with numerous positive effects on human health, encompasses tocotrienol-rich fraction (TRF), a natural variant abundant in palm oil.

**Objective:**

This systematic review analyzed findings from randomized controlled trials published until 2022 to evaluate the health impacts of palm TRF.

**Data Sources:**

A literature search was performed in Cochrane Central Register of Controlled Trials (CENTRAL), PubMed, OVID Medline, SCOPUS, and Web of Science from inception until December 2022. Thirty studies involving 2646 patients, including both healthy individuals and those with underlying conditions, were identified.

**Results:**

This review shows palm TRF to be a promising natural supplement against inflammation and lipid peroxidation and that can significantly enhance overall health. Additionally, the study underscores the necessity for further research to ascertain the optimal dosage, formulation, and duration of supplementation, maximizing the potential health advantages.

**Conclusion:**

This systematic review provides evidence supporting the health benefits associated with palm TRF.

**Systematic Review Registration:**

PROSPERO registration no. CRD42020204070.

## INTRODUCTION

Vitamin E, a vital micronutrient in lipid-rich plant products, has been extensively studied for its antioxidant properties. There are 2 naturally occurring forms of vitamin E—namely, tocopherol (Toc) and tocotrienol (T3). Both forms share a similar chemical structure, which consists of a 6-chromanol ring and a hydrophobic carbon side chain, which is saturated in Toc and unsaturated in T3.^1^ Each form can exist naturally in 4 isoforms—namely, alpha (α), beta (β), delta (δ), and gamma (γ).^2^^,^^3^ In recent years, there has been a surge of interest in the potential health benefits of T3 and its therapeutic uses. For instance, T3 was reported to possess superior antioxidant,^4^^,^^5^ anticancer,^6^ anti-inflammatory,^7^ neuroprotective,^8^ immunomodulatory,^9^^,^^10^ and antidiabetic properties^11–13^ compared with Toc. It has been suggested that the superior effects of T3 may be attributed to its unique chemical structure, which enables it to penetrate more efficiently into tissues with saturated fatty layers, such as the brain and liver, promoting efficient metabolic function.^2^^,^^14^

T3 is commonly found in various plant oils, including palm, rice bran, barley, wheat germ, annatto seeds, grains, and nuts.^15^ However, the concentration and isoforms of T3 in these natural sources vary significantly.^16^ The highest levels of T3 are found in rice bran oil,^3^ palm oil,^17^ and the seeds of annatto bean.^18^ The vitamin E derived from palm oil, known as the tocotrienol-rich fraction (TRF), contains a mixture of T3 isoforms (75%) and αToc (25%).^19^ TRF has the strongest potential for therapeutic use as it contains pure multiple isoforms of T3 and αToc.^19^

T3s are known to have short bioavailability, peaking at 3.5–4 hours in the plasma, before disappearing completely at 24 hours.^20^^,^^21^ T3s are hydrophobic, requiring a protein carrier for transport into the cell. Like other dietary lipids, absorption occurs in the small intestine and are incorporated into chylomicrons before entering the plasma.^21^^,^^22^ Evidence in humans has shown that T3 bioavailability differs from Toc in various organs, such as the brain, liver, skin, cardiac muscle fiber, and adipose tissue.^23^ In a study with human diploid fibroblasts, uptake of δ-T3 is the highest followed by γ-T3 and α-T3, respectively.^20^ Different preferential uptake of isoforms indicates different bioactivities; however, there is a need to identify these mechanisms to further enhance therapeutic benefits.

The use of TRF in clinical medicine is gaining recognition as evidenced by the growing number of randomized controlled trials (RCTs) that have demonstrated several health benefits,^3^ such as cardioprotective,^24^ neuroprotective,^8^ antioxidant,^4^^,^^5^^,^^25^^,^^26^ immunomodulatory,^9^^,^^10^ and anticholesterolemic properties.^7^^,^^27^^,^^28^ However, despite the compelling evidence from these RCTs, there has been a lack of a comprehensive review that summarizes the optimal dosage, duration, frequency of use, and potential therapeutic applications of TRF supplementation. Therefore, we conducted a study with the objective of providing a comprehensive analysis of the therapeutic and/or prophylactic intervention of palm TRF supplementation in an RCT setting.

## METHODS

The protocol for this systematic review was registered with PROSPERO (www.crd.york.ac.uk/PROSPERO; CRD42020204070). Reporting of this review was done in accordance with the Preferred Reporting Items for Systematic Reviews and Meta-Analyses (PRISMA) (Table S1).^29^

### Data sources and eligibility criteria

A systematic review search was conducted by A.D.L. in the following databases: PubMed, OVID Medline, CENTRAL, SCOPUS, and Web of Science from inception until December 2022 without using time restrictions. The inclusion criteria were as follows: (1) the study must be an RCT, (2) the study investigated health benefits, (3) the TRF used as a supplement was derived from palm oil, and (4) the paper was published in the English language. The PICOS (Population, Intervention, Comparison, Outcomes, Study design) strategy was used to define search strategies and establish eligibility criteria (Table 1).

**Table 1.** PICOS Criteria for Inclusion and Exclusion of Studies

Parameters	Criteria
Population	Humans (healthy or with comorbidities); no restriction on sex, race, or ethnicity
Intervention	Palm tocotrienol-rich fraction (must contain ≥3 T3s derived from palm oil)
Control/comparator	Placebos that do not contain any T3s
Outcomes	Health biomarkers
Study design	Randomized controlled trials

**Figure 1.**
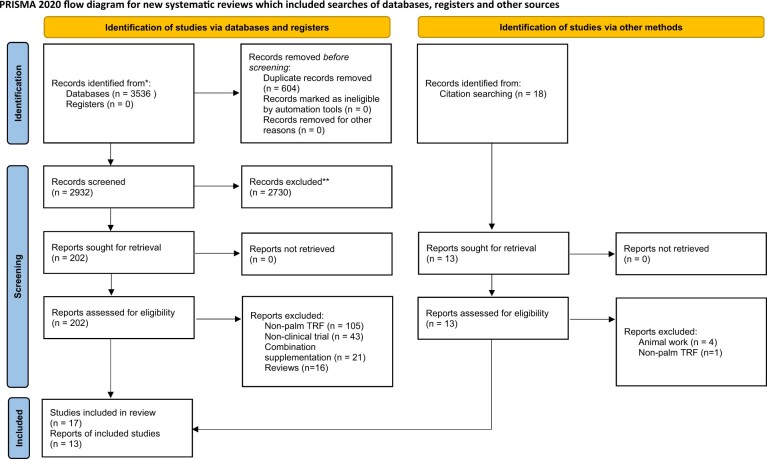
Database Search Results According to the Preferred Reporting Items for Systematic Reviews and Meta-Analyses (PRISMA) Statement.^29^ Abbreviation: TRF, tocotrienol-rich fraction

Strategic searches used the Boolean format: (“Vitamin E” OR “Tocopherol” OR “Tocotrienol” OR “Alpha-tocopherol” OR “Beta-tocopherol” OR “gamma-tocopherol” OR “delta-tocopherol OR “alpha-tocotrienol” OR “beta-tocotrienol” OR “gamma-tocotrienol” OR “delta-tocotrienol” OR “tocotrienol-rich fraction” OR “palm tocotrienol” OR “palm fruit” OR “palm oil”) AND (“randomized controlled trial” OR “RCTs”). Reference lists of selected articles were assessed to identify potential studies that were not included in the selected databases. The PRISMA 2020 chart workflow is shown in Figure 1.^29^ One reviewer (A.D.L.) initially screened the titles and abstracts of all retrieved articles. Two investigators (A.D.L. and A.K.R.) independently reviewed the selected articles to confirm. The full texts of eligible articles were then independently examined by 2 investigators (A.D.L. and A.K.R.) to choose those that fulfilled the selection criteria. Any disagreements on study selection were resolved by discussion with another investigator (U.D.P.).

### Data extraction and quality assessment

Two investigators (A.D.L. and A.K.R.) carried out the data extraction and confirmed the data independently. The titles and abstracts were screened for eligibility before the evaluation of the full text. Data extraction included study design and results. The effect sizes for each parameter, including mean changes and standard deviations of the intervention and control/groups, were extracted from each study. Authors of selected studies were contacted for further information when insufficient data were provided. The quality of each study was assessed using the Cochrane Collaboration tool for assessing the risk of bias (RoB).^30^ The Cochrane Collaboration tool consists of 5 domains: sequence generation, allocation concealment, blinding, incomplete outcome data, and selective outcome reporting. Each study was graded as having low risk of bias, high risk of bias, or unclear risk of bias for each domain. The risk of bias in each study was judged against each source as low, high, or some concern. The overall risk of bias for each outcome (across domains) within studies was rated as low risk of bias if all domains were assessed as being low risk of bias, whereas the outcome was rated as some concern of risk of bias if at least 1 domain is classified as having some concern with risk. The overall risk of bias was rated as high risk of bias if at least 1 domain was judged as being at high risk. Discussion with a third investigator (U.D.P.) was used to resolve any disagreements between A.D.L. and A.K.R. when the risk of bias analysis was carried out. The risk of bias was evaluated by using Review Manager version 5.4. For data synthesis and analysis, the outcomes of interest were the health biomarkers at the end of the study intervention.

## RESULTS

### Search results and study characteristics

A total of 3536 articles were identified from the databases, as shown in the PRISMA chart (Figure 1). Following the removal of duplicates and screening of the titles and abstracts, a total of 63 texts and their references were screened. A total of 30 studies met the inclusion criteria and were included in the systematic review (Figure 1). Characteristics of all studies investigated in this systematic review are provided in Table 2.^4^^,^^5^^,^^7–10^^,^^12^^,^^13^^,^^24–28^^,^^31–48^ All 30 studies were published from 1995 to 2022. The majority (25) of the clinical trials were conducted in Malaysia (Table 2), 4 were conducted in the United States,^27^^,^^28^^,^^34^^,^^45^ and 1 study was conducted in Australia.^37^ The study designs used in these clinical trials include placebo-controlled, double-blinded, crossover RCTs (2/30),^28^^,^^43^ placebo-controlled, single-blinded, parallel RCTs (2/30),^10^^,^^26^ placebo-controlled, double-blinded, 3-arm, parallel RCTs (3/30),^5^^,^^31^^,^^37^ and placebo-controlled, double-blinded, parallel RCTs (23/30).^4^^,^^7–9^^,^^12^^,^^13^^,^^24^^,^^25^^,^^27^^,^^33–35^^,^^38–42^^,^^44–48^

**Table 2.** Study Characteristics of Patients and Interventions

							Intervention		Outcome	
**No.**	**First author (year)**	**Location**	**Patient characteristics**	**TRF content per capsule**	**Brand**	**Treatment duration**	**Intervention**	**Placebo**	**Intervention**	**Placebo**
Healthy										
1	Azman et al (2008)^5^	Malaysia	Age: >50-55 y M:F = 26:45 *n* = 71 Healthy	Tocotrienol (74%) α-T3 - 70.4 mg β-T3 - 4.8 mg γ-T3 - 57.6 mg δ-T3 - 33.6 mg Tocopherol (26%) α-Toc – 48 mg	Gold TriE tocotrienol; Sime Darby Bioorganic	6 mo	Treatment 1: 150 mg TRF capsule per day Treatment 2: α-Toc 400 IU capsule per day	Olive oil capsule	**Significant improvements ( *P* < .05):** SOD ↑^a^ GSSG ↓^a^ GPX (female participants) ↑^a^ **No significant improvements:** GSH Catalase	**Significant changes ( *P* < .05):** Catalase (placebo) ↓^a^
2	Goon et al (2017)^4^	Malaysia	Age: 52.2 ± 2.1 y M:F = 26:45 *n* = 71 Healthy	Tocotrienol (74%) α-T3 - 70.4 mg β-T3 - 4.8 mg γ-T3 - 57.6 mg δ-T3 - 33.6 mg Tocopherol (26%) α-Toc - 48 mg	Sime Darby Food and Beverages Marketing Sdn Bhd	6 mo	Treatment 1: 150 mg TRF Treatment 2: 1 α-Toc capsule per day	Olive oil capsule	Significant improvement ( *P* < .05): Malonaldehyde - TRF group ↓^a^ Carbonyl content - Toc group ↓^a^ DNA damage - TRF group ↓^a^ Vitamin D concentration^a^ (TRF and Toc group) ↑	Significant changes ( *P* < .05): Malonaldehyde ↑^a^
3	Chin et al (2008)^25^	Malaysia	Age: 35-49 and >50 y M:F = 0:62 *n* = 62 Healthy	Tocotrienol (74%) α-T3 - 70.4 mg β-T3 - 4.8 mg γ-T3 - 57.6 mg δ-T3 - 33.6 mg Tocopherol (26%) α-Toc - 48 mg	TriE tocotrienol; Golden Hope Plantation	6 mo	160 mg TRF daily	Placebo capsules	Significant improvement ( *P* < .05): DNA damage (TRF group) ↓^a^ Sister chromatic exchange frequency (TRF, >50 y) ↓^a^ 8-OHdG (TRF) ↓ No significant improvement	No significant changes
4	Ghani et al (2019)^26^	Malaysia	Age: 50-55 y M:F = 26:45 *n* = 71 Healthy	Tocotrienol (74%) α-T3 - 70.4 mg β-T3 - 4.8 mg γ-T3 - 57.6 mg δ-T3 - 33.6 mg Tocopherol (26%) α-Toc - 48 mg	Gold TriE tocotrienol; Sime Darby Bioorganic	6 mo	Treatment 1: 150 mg TRF capsule per day Treament 2: α-Toc 400 IU capsule per day	Olive oil capsule	Significant improvements ( *P* < .05): 3 mo (TRF) Aging (M) ↑ Cell division (M) ↓ Regulation of transcription (M) ↓ G-protein–coupled receptor signaling (M, F) ↓ Protein kinase activity (F) ↓ Multicellular organismal growth (F) ↓ Response to glucocorticoid (F) ↓ Cell surface receptor signaling (F) ↓ 6 mo (TRF) Integrin-mediated signaling (M) ↓ ERK1/2 (F/M) ↓ G-protein-coupled receptor (F) ↓ Cell surface receptor signaling apoptosis (F) ↓ I-kappa B kinase–nuclear factor-kappa B (F) ↓ Cell surface receptor signaling (F) ↓ Multicellular organismal growth (M, F) ↑	No significant improvements
5	Mahalingam et al (2011)^9^	Malaysia	Age: 18-25 y M:F = 0:108 *n* = 108 Healthy induced with a vaccine	Tocotrienol α-T3 - 61.52 mg γ-T3 - 112.8 mg δ-T3 - 25.68 mg Tocopherol α-Toc - 91.6 IU	Tocovid SupraBio, manufactured by Hovid Sdn Bhd (Ipoh, Malaysia)	8 wk	400 mg TRF daily	Two soy oil placebo capsules	Significant improvement ( *P* < .05): IFN-γ ↑ IL-4 ↑ IL-6↓ Anti-TT IgG ↑	No significant changes
6	Radhakrishnan et al (2008)^31^	Malaysia	Age: 20-50 y M:F = 19:34 *n* = 48 Healthy	Tocotrienol α-T3 - 113 mg β-T3 - 10 mg γ-T3 - 91 mg δ-T3 - 36 mg	Tocovid SupraBio; manufactured by Hovid Sdn Bhd (Ipoh, Malaysia)	8 wk	Treatment 1: 200 mg TRF Treatment 2: 200 mg α-Toc capsules per day	One placebo capsule per day	No significant improvements: IL-4 IFN-γ T-helper Cytotoxic T-lymphocyte B-lymphocyte NK cells	No significant changes
7	Jubri et al (2013)^10^	Malaysia	Age: 20-50 y M:F = 114:0 *n* = 114 Healthy smokers and nonsmokers	Tocotrienol 60% α-T3 γ-T3 δ-T3 Tocopherol 40% α-Toc	Source: Palmvitee Palm Oil Research Institute of Malaysia, now known as Malaysian Palm Oil Board (PORIM/ MPOB)	24 wk	Smoker group: Treatment: 200 mg TRF Nonsmoker group: Treatment: 200 mg TRF	Smoker group: One placebo capsule per day Nonsmoker group: One placebo capsule per day	Significant improvement ( *P* < .05): Lymphocyte proliferation ↑ CD4+ (smoking group) ↑ B cells (nonsmoking group) ↑ CD4+/CD8+ ratio increased (smoking group) ↑	No significant changes
8	Chin et al (2011)^32^	Malaysia	Age: 35-49 and >50 y M:F = 0:62 *n* = 62 Healthy	Tocotrienol (74%) α-T3 - 70.4 mg β-T3 - 4.8 mg γ-T3 - 57.6 mg δ-T3 - 33.6 mg Tocopherol (26%) α-Toc - 48 mg	Tri E tocotrienol; Golden Hope Plantation	6 mo	Treatment 1: 35-49 y old; 160 mg per day Treatment 2: >50 y old; 160 mg per day	Placebo capsule for both age groups	Significant improvement: SOD ↓^a^ GPX ↑^a^ Carbonyl content ↓^a^ Advanced glycosylation product ↓^a^ HDL-C (TRF; 35-49 y and >50 y) ↑^a^ HDL-C/TC (TRF; 35-49 y and >50 y) ↑^a^ No significant improvement: MDA TC LDL-C Triglycerides Catalase	Significant changes ( *P* < .05): GPX ↓^a^ Catalase (3 and 6 mo) ↓^a^ Triglycerides ↓^a^ HDL-C (>50-y group) ↓^a^ HDL-C/TC (>50-y group) ↓^a^
9	Rasool et al (2006)^33^	Malaysia	Age: 21-30 y M:F = 36:0 *n* = 36 Healthy	Tocotrienol (73.8%) α-T3 - 34.6% γ-T3 - 24.63% δ-T3 - 15% Tocopherol (26.2%) α-Toc - 26.2%	Tri E tocotrienol; Golden Hope Plantation	8 wk	Treatment 1: 80-mg capsule per day Treatment 2: 160-mg capsule per day Treatment 3: 320-mg capsule per day	Inert corn flour placebo	Significant improvement: Aortic BSP – 160-mg and 320-mg group ↓ Plasma TAS - 320 mg ↑ AI - 160 mg ↓ No significant improvement: PWV TC LDL-C	No significant changes
10	Rasool et al (2008)^24^	Malaysia	Age: 23 ± 0.39 y M:F = 36:0 *n* = 36 Healthy adults	Tocotrienol (76.5%) α-T3 - 23.54% γ-T3 - 43.16% δ-T3 - 9.83% Tocopherol (23.5%) α-Toc - 23.5%	Tocovid Suprabio; Hovid Bhd, Malaysia	8 wk	Treatment 1: 50-mg capsule per day Treatment 2: 100-mg capsule per day Treatment 3: 200-mg capsule per day	Soybean oil	Significant improvement: PWV - 100 mg and 150 mg ↓ AI - 50 mg, 100 mg, and 150 mg ↓ No significant improvement SBP DBP TC LDL-C	No significant changes
11	Mustad et al (2002)^34^	USA	Age: 40-47 y M:F = 39:29 *n* = 67 Healthy adults	Tocotrienol (74%) α-T3 - 61.52 mg β-T3 - 9.7 mg γ-T3 - 112.8 mg δ-T3 - 25.68 mg Tocopherol (26%) α-Toc - 78 mg	Tocovid Suprabio; Hovid Sdn Bhd, Malaysia	7 wk	Three different brands: Treatment 1 200 mg TRF Treatment 2 γ-T3 Treatment 3 P25-complex	1 g safflower oil (Solgar Laboratories, Leonia, NJ)	Significant change: TC (TRF) ↑ LDL-C (TRF) ↑ No significant improvement: HDL Triacylglycerol Glucose 8-epi-PGF	No significant changes
12	Mahdy et al. (2015)^35^; Aminuddin et al (2021)^36^ (same authors, study revisited)	Malaysia	Age: 25-26 y M:F = 0:299 *n* = 299 Pregnancy-induced hypertension (PIH)	Tocotrienol (70%) Tocopherol (30%)	TriE tocotrienol; Golden Hope Plantation	40 wk/12-16 wk into pregnancy until end of gestation	100 mg	Super olein oil capsules without TRF	Significant improvement ( *P* < .05): PIH incidence rate ↓ Pre-eclampsia incidence rate ↓	No significant changes
Comorbidities										
13	Daud et al (2013)^27^	USA	Age: 58 ± 13 y M:F = 43:38 *n* = 81 End-stage renal disease	Tocotrienol (82%) α-T3 - 30.18 mg β-T3 - 5.30 mg γ-T3 - 41.66 mg δ-T3 - 12.86 mg Tocopherol (18%) α-Toc - 20 mg	Carotino Sdn Bhd, Malaysia	16 wk	180 mg TRF	Placebo capsules	Significant improvement ( *P* < .05): Plasma triacylglycerol (TRF) ↓ HDL-C (TRF) ↑ TC (TRF and placebo) ↑ LDL-C (TRF and placebo) ↓ Plasma apoliprotein A1 (TRF) ↑ Cholesteryl-ester transfer (TRF) ↑ No significant improvement: CRP IL-6 TAP TBARS	No significant changes
14	Stonehouse et al (2016)^37^	Australia	Age: 53-65 y M:F = 36:22 *n* = 90 Type 2 diabetes	Tocotrienol (77%) α-T3 - 67.6 mg β-T3 - 9.7 mg γ-T3 - 97.7 mg δ-T3 – 35 mg Tocopherol (23%) α-Toc- 66 mg	Carotino Sdn Bhd, Malaysia	8 wk	420 mg TRF	Two palm olein capsules per day	No significant improvement: hsCRP IL-6 TNF-α ICAM-1 VCAM-1 E-selectin PAI-1 SBP DBP Adiponectin TC HDL-C LDL-C TG TC/HDL-C ratio HbA1c Insulin HOMA2-IR Glucose	No significant changes
15	Tan et al (2018)^38^	Malaysia	Age: 61.6 ± 9.5 y M:F = 48:18 *n* = 66 Type 2 diabetes	Tocotrienol α-T3 - 61.52 mg β-T3 - 9.7 mg γ-T3 - 112.8 mg δ-T3 - 25.68 mg Tocopherol α-Toc - 78 mg	Tocovid Suprabio; Hovid Bhd, Malaysia	8 wk	400 mg TRF	Two placebo capsules	Significant improvement: Creatinine ↓ No significant improvement: HbA1c SBP DBP UACR AGE sRAGE Ne-CML Cystatin	No significant changes
16	Tan et al (2019)^13^	Malaysia	Age: 49-74 y M:F = 35:19 *n* = 54 Type 2 diabetes	Tocotrienol α-T3 - 61.52 mg γ-T3 - 112.8 mg δ-T3 - 25.68 mg Tocopherol α-Toc - 91.60 IU	Tocovid Suprabio; Hovid Bhd, Malaysia	12 wk	400 mg TRF	Two placebo capsules per day	Significant improvement: eGFR ↑ Urea ↑ Creatinine ↓ VCAM-1 ↓ No significant improvement: TNFR-1 HbA1c SBP DBP UACR Uric acid MDA	No significant improvements
17	Ng et al (2020)^39^	Malaysia	Age: 63 ± 14 y M:F = 52:28 *n* = 77 Type 2 diabetes	Tocotrienol α-T3 - 61.52 mg γ-T3 - 112.8 mg δ-T3 - 25.68 mg Tocopherol α-Toc - 91.60 IU	Tocovid Suprabio; Hovid Bhd, Malaysia	8 wk	400 mg TRF	One placebo capsule per day	Significant improvement ( *P* < .05): Peak velocity (median, sural) ↑ Conduction velocity (median, sural, tibial) ↑ PP amplitude (median) ↑ NGF ↑ TC ↑ No significant improvement: NGF MDA VCAM-1 TNFR-1 TXB2 HbA1c SBP DBP eGFR Urea AST ALT HDL-C	No significant changes
18	Koay et al (2021)^12^	Malaysia	Age: 67 ± 14 y M:F = 38:21 *n* = 59 Type 2 diabetes	Tocotrienol α-T3 - 61.52 mg γ-T3 - 112.8 mg δ-T3 - 25.68 mg Tocopherol α-Toc - 91.60 IU	Tocovid Suprabio; Hovid Bhd, Malaysia	1 y	400 mg TRF	Placebo capsules	Significant improvement ( *P* < .05): Creatinine ↓ eGFR NGF ↑ Urea ↑ No significant improvement: TGF-β1 VEGF-A HbA1c SBP DBP UACR Uric acid TGF-β1 VEGF-A	No significant changes
19	Chiew et al (2021)^41^	Malaysia	Age: 61.51 ± 1.25 y M:F = 32:11 *n* = 43 Type 2 diabetes	Tocotrienol α-T3 - 61.52 mg γ-T3 - 112.8 mg δ-T3 - 25.68 mg Tocopherol α-Toc - 91.60 IU	Tocovid Suprabio; Hovid Bhd, Malaysia	8 wk	400 mg TRF	Placebo capsules	Significant improvement ( *P* < .05): AST ↓ ALT ↓ Retinal hemorrhage, right eye No significant improvement: HbA1c AGE sRAGE Ne-CML Cystatin C	No significant changes
20	Chuar et al (2021)^40^	Malaysia	Age: 64 ± 13 y M:F = 58:30 *n* = 88 Type 2 diabetes	Tocotrienol (77%) α-T3 - 61.52 mg γ-T3 - 112.8 mg δ-T3 - 25.68 mg Tocopherol (23%) α-Toc - 61.1 mg	Tocovid Suprabio; Hovid Bhd, Malaysia	6-mo intervention followed by 6-mo washout	400 mg	400-mg placebo capsules	Significant improvement ( *P* < .05): PP amplitude (sural sensory nerve) ↑ Conduction velocity (median sensory nerve, sural sensory nerve, tibial motor nerve) ↑ PP amplitude (median sensory nerve, sural sensory nerve) ↑ No significant improvement: HbA1c SBP DBP TGF-β1 VEGF-A eGFR Serum creatinine Urea	No significant changes
21	Hor et al (2018)^42^	Malaysia	Age: 57.6 ± 8.9 y M:F = 130:170 *n* = 300 Type 1 and type 2 diabetes	Tocotrienol α-T3 - 61.52 mg γ-T3 - 112.8 mg δ-T3 - 25.68 mg Tocopherol α-Toc - 91.60 IU	Tocovid Suprabio; Hovid Bhd, Malaysia	1 y	400 mg	Two placebo capsules	Significant improvements ( *P* < .05): Lancinating pain score HbA1c >8.0% ↓ NIS (6 and 12 mo) ↓^a^ Significant changes ( *P* < .05) Homocysteine ↑ ^a^ No significant improvement: HbA1c FBC Total Symptoms Score (TSS)	Significant changes ( *P* < .05): NIS^a^ (6 and 12 mo) ↓ Homocysteine ↑ ^a^
22	Heng et al (2015)^7^	Malaysia	Age: 20-60 y M:F = 20:37 *n* = 57 Metabolic syndrome	Tocotrienol (77%) α-T3 - 61.52 mg γ-T3 - 112.8 mg δ-T3 - 25.68 mg Tocopherol (23%) α-Toc - 61.1 mg	Tocovid Suprabio; Hovid Bhd, Malaysia	16 wk	400 mg TRF	Soybean oil capsule	Significant change ( *P* < .05): TC (TRF) ↓ LDL-C (TRF) ↓ HDL-C (TRF) ↓ TC/HDL (TRF) ↑ IL-6 (TRF) ↓ TNF-α (TRF) ↓ No significant change: SBP DBP TG LDL/HDL FPG CRP Adiponectin Leptin	No significant changes
23	Gan et al (2017)^43^	Malaysia	Age: 26-52 y M:F = 15:16 *n* = 31 Metabolic syndrome	Tocotrienol (77%) α-T3 - 61.52 mg γ-T3 - 112.8 mg δ-T3 - 25.68 mg Tocopherol (23%) α-Toc - 91.60 IU	Tocovid Suprabio; Hovid Bhd, Malaysia	2 wk	400 mg TRF	One palm olein capsule per day	No significant improvement: hsCRP P-selectin ICAM-1 VCAM-129 D-dimer Activated glycoprotein Iib/IIIa receptor	No significant changes
24	Qureshi et al (1991)^28^	Malaysia	Age: 30-60 y M:F =14:11 *n* = 25 High cholesterol levels	50 mg TRF: Tocotrienol α-T3 – 12%-15% γ-T3 – 35%-40% δ-T3 – 25%-30% *Tocopherol* α-Toc – 15%-20%	Palmvitee; Palm Oil Research Institute of Malaysia (PORIM/MPOB)	4 wk	200 mg TRF	Corn oil capsule	Significant change ( *P* < .05): TC (TRF) ↓ LDL-C (TRF) ↓ Apoliprotein B (TRF) ↓ HDL-C (TRF) ↑ No significant improvement: Apolipoprotein A	No significant changes
25	Gopalan et al (2014)^8^	Malaysia	Age: 52 ± 8.5 y M:F = 96:146 *n* = 242 CVD risk	Tocotrienol α-T3 - 61.52 mg γ-T3 - 112.8 mg δ-T3 - 25.68 mg Tocopherol α-Toc - 91.60 IU	Tocovid Suprabio; Hovid Bhd, Malaysia	2 y	200 mg TRF	One palm olein capsule per day	Significant improvement ( *P* < .05): Mean white matter lesion (TRF) ↑ No significant improvement: hsCRP TC HDL LDL TG Apolipoprotein B Lipoprotein A	No significant changes
26	Magosso et al (2013)^44^	Malaysia	Age: 42-61 y M:F = 34:53 *n* = 87 NAFLD	Tocotrienol α-T3 - 61.52 mg γ-T3 - 112.8 mg δ-T3 - 25.68 mg Tocopherol α-Toc - 61.1 mg	Tocovid Suprabio; Hovid Bhd, Malaysia	1 y	200 mg TRF	One palm olein capsule per day	Significant improvement ( *P* < .05): Hepatic echogenic response ↑ No significant improvement: hsCRP TC HDL LDL Triglyceride ALP AST GGT Apolipoprotein B Lipoprotein A Serum creatinine SBP DBP	No significant changes
27	Tomeo et al (1995)^45^	Malaysia	Age: 49-89 y M:F = 23:27 *n* = 50 Carotid artery atherosclerosis	Tocotrienol (40mg) α-T3 γ-T3 Tocopherol α-Toc - 16 mg	Palmvitee; Palm Oil Research Institute of Malaysia (PORIM/MPOB)	18 mo	First 3 mo: 4 capsules (160 mg) After third month: 6 capsules (240 mg)	Placebo First 3 mo: 4 capsules After third month: 6 capsules	Significant improvement ( *P* < .05): Collagen-induced platelet aggregation responses ↑ No significant improvement ( *P* < .05): HDL-C Cholesterol LDL-C Triglyceride Plasma HDL-C	No significant changes
28	Yuen et al (2011)^46^	Malaysia	Age: 57.6 ± 8.9 y M:F = 20:12 *n* = 32 Hypercholesterolemic	Tocotrienol α-T3 - 15.4 mg γ-T3 - 28.2 mg δ-T3 - 6.4 mg Tocopherol α-Toc - 22.9 IU	Tocovid Suprabio; Hovid Bhd, Malaysia	6 mo	300 mg	300 mg soya bean oil	Significant improvement ( *P* < 0.05): TC ↓ LDL-C ↓ TBARS ↓ No significant improvement: Triacylglycerol HDL-C	Significant changes ( *P* < .05): Collagen (12 mo) ↑ TBARS (12 mo) ↑
29	Musa et al (2022)^47^	Malaysia	Age: 60.88 ± 7.79 y M:F = 201:49 *n* = 250 Post-atrial fibrillation with medical conditions	Tocotrienol (77%) α-T3 - 61.52 mg γ-T3 - 112.8 mg δ-T3 - 25.68 mg Tocopherol (23%) α-Toc - 61.1 mg	Tocovid Suprabio; Hovid Bhd, Malaysia	6 wk	400 mg	400 mg palm super olein	Significant improvement ( *P* < .05): 36-item Short Form Survey: Role physical Role emotional Nottingham Health Profile: Sleep quality No significant improvement: Postoperative outcomes Postoperative stay Health-related quality of life	No significant changes
30	Tan et al (2016)^48^	Malaysia	Age: 9.4 ± 1.9 y M:F = 124:22 *n* = 146 ADHD	Tocotrienol α-T3 - 30.76 mg γ-T3 - 56.40 mg δ-T3 - 12.84 mg Tocopherol α-Toc - 45.80 IU	Tocovid Suprabio; Hovid Bhd, Malaysia	6 mo	200 mg	Two placebo capsules	Significant improvement ( *P* < .05): VAPRS (3 mo and 6 mo) No significant improvement: VATRS	No significant changes

*Abbreviations:* ADHD, attention-deficit/hyperactivity disorder; AGE, advanced glycation end-products; AI, Augmentation Index; ALP, alkaline phosphatase; ALT, alanine transaminase; AST, aspartate transaminase; CRP, C-reactive protein; DBP, diastolic blood pressure; eGFR, estimated glomerular filtration rate; FBC, fasting blood glucose; FPG, fasting plasma glucose; GGT, Gamma-glutamyl transferase; GPX, glutathione peroxidase; GSSG, glutathione disulfide; HDL-C, high-density-lipoprotein cholesterol; HbA1c, glycated hemoglobin; hsCRP, high-sensitivity C-reactive protein; HOMA2-IR, homeostasis model assessment of insulin resistance; ICAM, intercellular adhesion molecule; IFN, interferon; IL, interleukin; LDL-C, low-density-lipoprotein cholesterol; MDA, malondialdehyde; Ne-CML, Ne-carboxymethyl lysine; NGF, nerve growth factor; NIS, Neuropathy Impairment Score; PAI-1, Plasminogen activator inhibitor-1; PIH, pregnancy-induced hypertension; PP, peak-to-peak; PWV, pulse wave velocity; SBP, systolic blood pressure; SOD, superoxide dismutase; sRAGE, soluble receptor for advanced glycation products; T3, tocotrienol; TAS, total antioxidant status; TBARS, thiobarbituric acid reactive substances; TC, total cholesterol; TG, triacylglycerol; TGF-β, transforming growth factor beta; TNF-α, tumor necrosis factor-alpha; TNFR, tumor necrosis factor receptor; Toc, tocopherol; TRF, tocotrienol-rich fraction; TXB2, Thromboxane 2; VCAM, vascular cell adhesion protein 1; UACR, urine albumin to creatinine ratio; VAPRS, National Institute for Children Health Quality (NICHQ) Vanderbilt ADHD Parent Rating Scale; VATRS, National Institute for Children Health Quality (NICHQ) Vanderbilt ADHD Teacher Rating Scale; VEGF, vascular endothelial growth factor; α-Toc, alpha-tocopherol; α-T3, alpha-tocotrienol; β-T3, beta-tocotrienol; δ-T3, delta-tocotrienol; γ-T3, gamma-tocotrienol; 8-OHdG, 8-hydroxyguanosine.

a Indicative of statistical comparison between baseline and endpoint within the same group. Its absence indicates statistical comparison between the endpoint of the placebo and the endpoint of the intervention.

A total of 2626 participants were investigated in these studies. Eleven studies (*n* = 746) included healthy participants,^4^^,^^5^^,^^9^^,^^10^^,^^24–26^^,^^31–34^ while 19 studies (*n* = 1880) included patients who had morbidities.^7^^,^^8^^,^^12^^,^^27^^,^^28^^,^^36–40^^,^^42–48^ In 1 of the studies in healthy participants, the healthy volunteers recruited for the study were given a vaccine (tetanus toxoid) as the immunological challenge to induce an immune response.^9^ In another study, the researchers compared the effects of TRF supplementation in smokers and nonsmokers.^10^ In the groups with morbidities, 1 study investigated end-stage renal disease,^27^ 1 study investigated attention-deficit/hyperactivity disorder (ADHD),^48^ 1 study investigated pregnancy-induced hypertension (PIH),^35^^,^^36^ 1 study investigated postoperative atrial fibrillation,^47^ 2 studies investigated metabolic syndrome,^7^^,^^43^ 5 studies investigated the risk of cardiovascular disease (CVD),^8^^,^^28^^,^^44–46^ and 8 studies investigated diabetes mellitus.^12^^,^^13^^,^^37–42^ The age of the participants/patients ranged from 7 to 70 years. While most studies investigated the effects of TRF in adults, only 1 study^48^ investigated the effect of TRF supplementation in children.

The dosage of TRF used in these clinical studies varied from 50 to 420 mg daily. For instance, in 2 studies, participants received daily supplementation of 100 mg TRF^28^^,^^35^; in 3 studies, the participants were supplemented with 150 mg TRF^4^^,^^5^^,^^26^; and in 2 studies, the participants were supplemented with 160 mg TRF.^25^^,^^32^ In 1 study, the participants were supplemented with 180 mg TRF,^27^ 300 mg TRF,^46^ and 420 mg TRF.^37^ There were 17 studies in which the participants were supplemented with 200 mg TRF^8^^,^^31^^,^^34^^,^^44^^,^^48^ or 400 mg TRF.^7^^,^^9^^,^^10^^,^^12^^,^^13^^,^^38–43^^,^^47^ In 3 studies, the participants were given different doses of TRF. In 1 study, the participants were supplemented with 50 mg, 100 mg, or 200 mg of TRF,^33^ whereas in another study, the participants were given 80 mg, 160 mg, or 320 mg of TRF.^24^ In contrast, Tomeo et al^45^ varied the supplementation in which the participants received 120 mg TRF for the first 3 months of the study and 240 mg for the remainder of the study.

All studies reported on the levels of all TRF isomers found in the blood. However, 2 studies^10^^,^^45^ did not analyze the T3 isoforms. All of the studies included in this systematic review used TRF sourced from palm oil. The TRF intervention used was manufactured by 4 nutraceutical companies. Tocovid Suprabio, used in 18 studies, is manufactured by Hovid Sdn Bhd,^7–9^^,^^12^^,^^13^^,^^24^^,^^31^^,^^34^^,^^38–44^^,^^46–48^ Tri-E T3, used in 7 studies, is manufactured by Sime Darby,^4^^,^^5^^,^^25^^,^^26^^,^^32^^,^^33^^,^^35^ Palmvitee, used in 3 studies, is made by the Malaysian Palm Oil Board,^10^^,^^28^^,^^45^ and 2 studies used TRF made by Carotino Sdn Bhd.^27^^,^^37^ All studies reported the use of a placebo in their study. Thirteen studies did not report on the content of the placebos used in the study.^10^^,^^12^^,^^13^^,^^25^^,^^27^^,^^31^^,^^32^^,^^38–42^^,^^45^

The duration of the interventions ranged from 2 weeks to 2 years. For instance, 1 study used a 2-week intervention duration,^43^ while others used intervention durations of 4 weeks,^28^ 6 weeks,^47^ 7 weeks,^34^ 8 weeks,^9^^,^^24^^,^^31^^,^^33^^,^^37–39^^,^^41^ 12 weeks,^13^ 16 weeks,^7^^,^^27^ 6 months,^4^^,^^5^^,^^10^^,^^25^^,^^32^^,^^40^^,^^46^^,^^48^ 10 months,^35^ 1 year,^12^^,^^44^^,^^46^ 18 months,^45^ or 2 years.^8^

**Figure 2.**
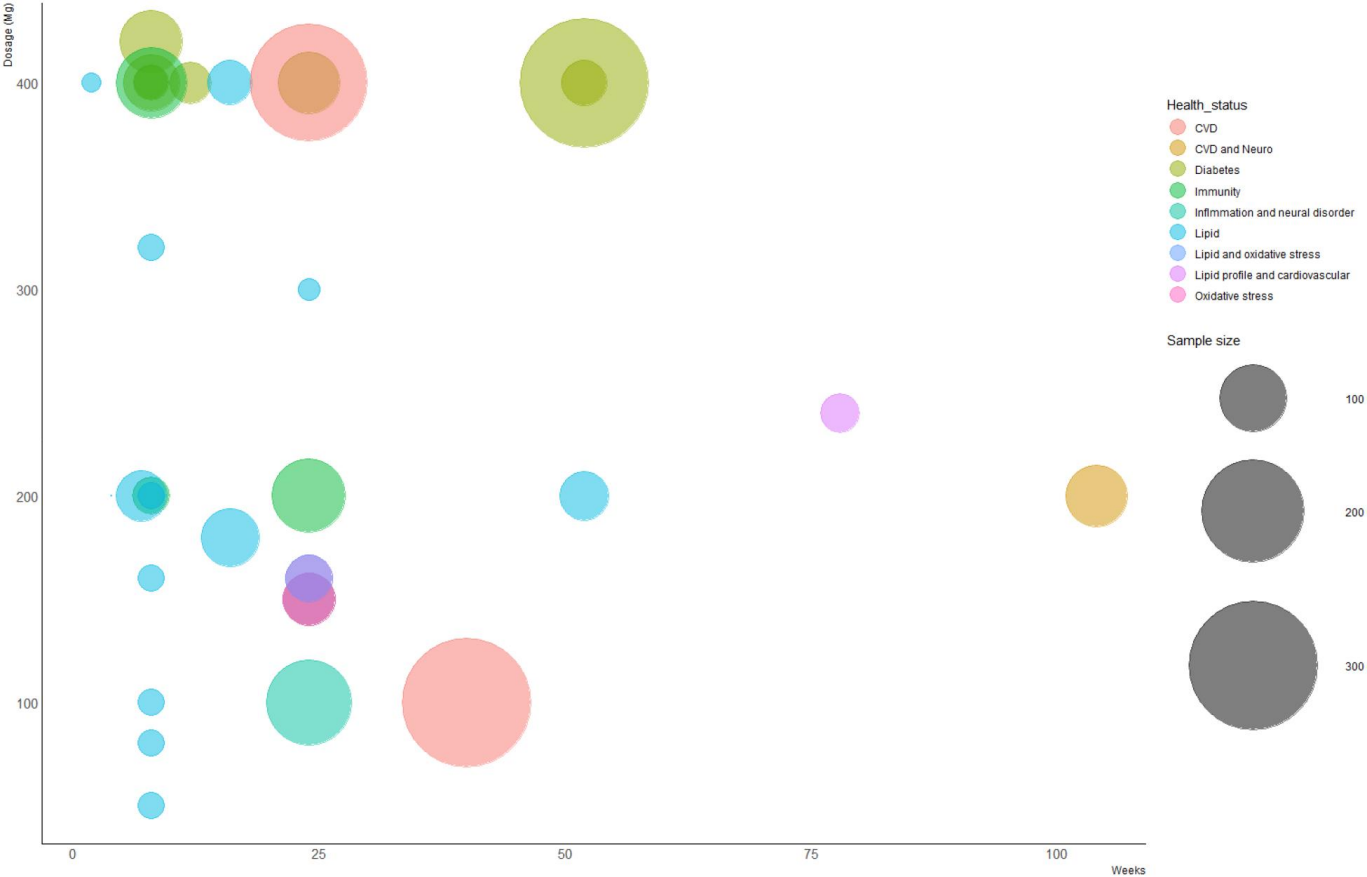
Bubble Chart Showing Duration in Weeks of Study Intervention Against Dosage Used. The size of the bubble represents the number of individuals in a study, and the color represents the health status of the patients. The majority of the studies investigated TRF interventions in doses that were equal to or more than 400 mg and doses that were equal to or less than 200 mg. Abbreviations: CVD, cardiovascular disease; Neuro, neurology; TRF, tocotrienol-rich fraction

The trials measured different parameters on the effects of TRF on health biomarkers, such as oxidative stress biomarkers,^4^^,^^5^^,^^25^ immunological responses,^9^^,^^10^^,^^26^^,^^31^ cardiometabolic markers,^7^^,^^8^^,^^12^^,^^13^^,^^24^^,^^27^^,^^28^^,^^32–35^^,^^37^^,^^39–46^^,^^48^ and neurodevelopmental disorders,^48^ and post-cardiac surgery.^47^ A bubble chart presenting dosage, population, and duration of TRF supplementation and health biomarkers with corresponding study size is shown in Figure 2.

### Quality assessment of studies

**Figure 3.**
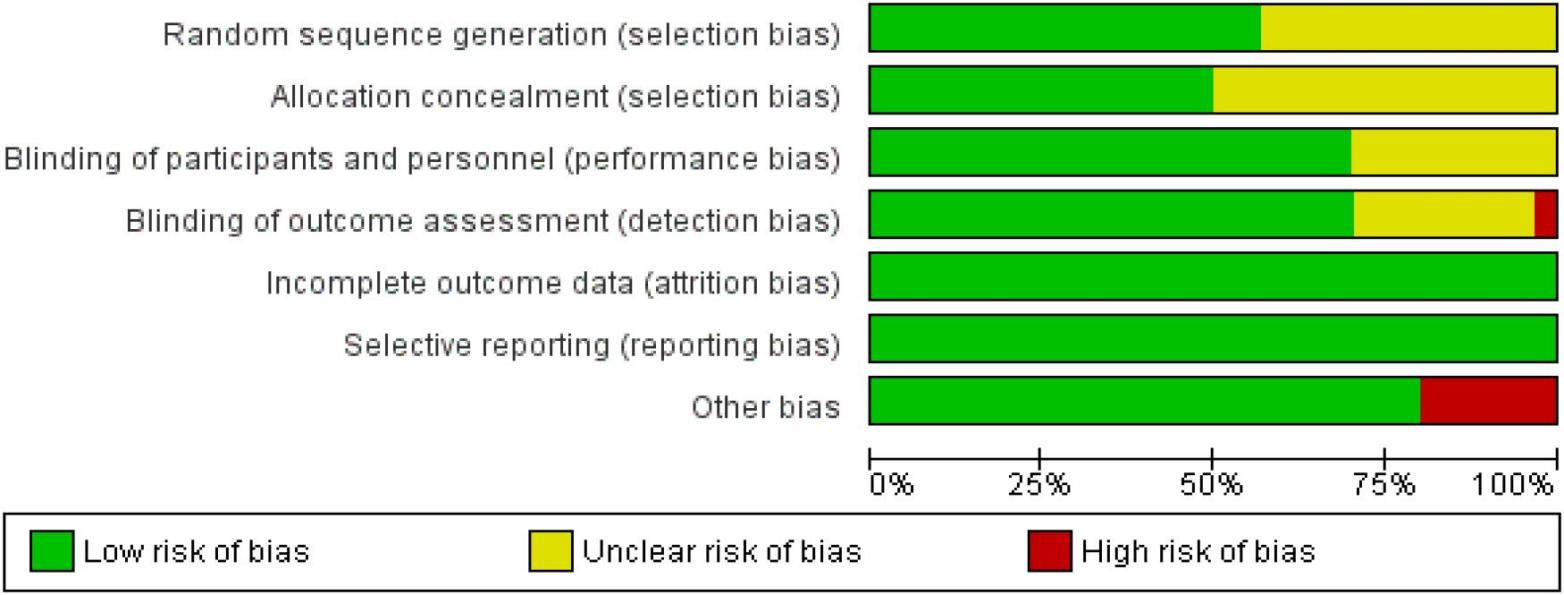
An Overview of the Risk of Bias of All the Included Studies

**Figure 4.**
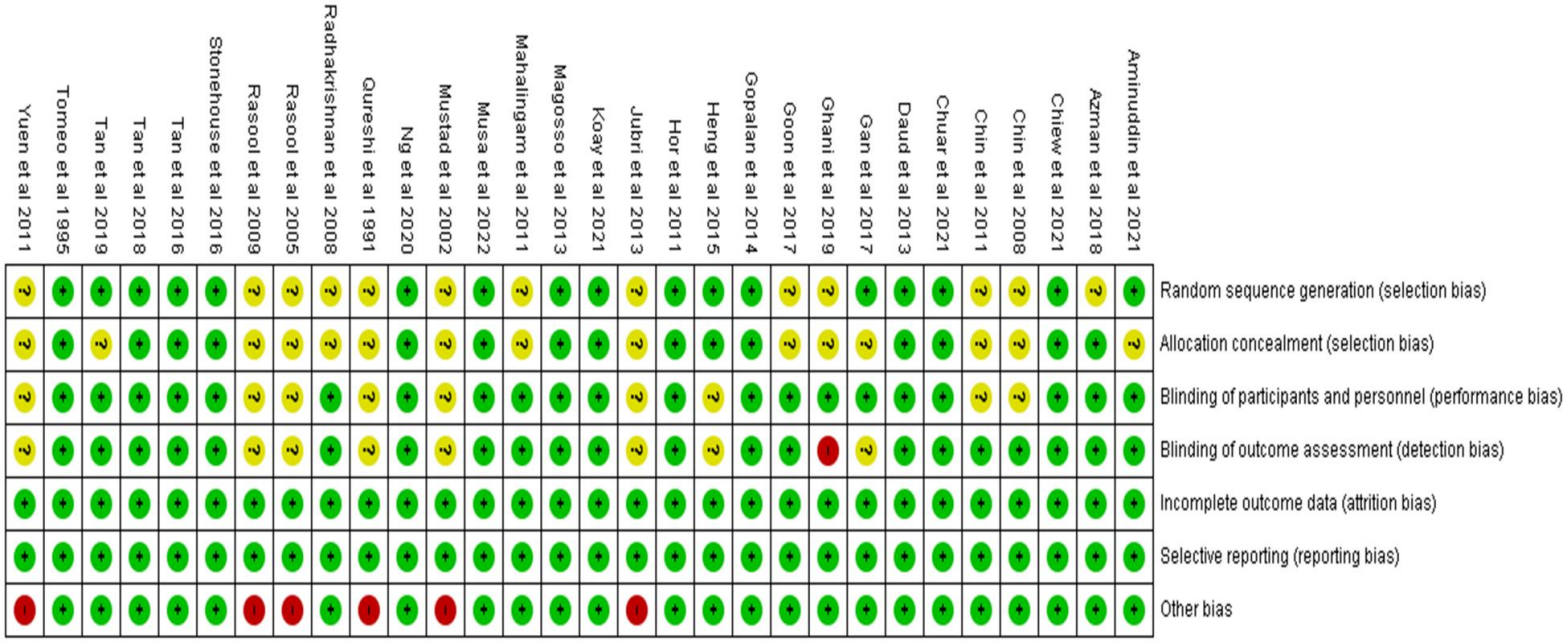
Risk of Bias for Each Study

The quality of each study included in this systematic review is summarized in Figures 3 and 4. Fifteen (15/30) studies were rated as low risk of bias in all of the domains based on Cochrane's RoB criteria. The random-sequence generation (selection bias) was not clearly reported in 13 studies.^4^^,^^5^^,^^9^^,^^10^^,^^24–26^^,^^28^^,^^31–34^^,^^46^ The allocation concealment was not clearly reported in 15 studies.^4^^,^^9^^,^^10^^,^^13^^,^^24–26^^,^^28^^,^^31–35^^,^^43^^,^^46^ The blinding of patients was not clearly reported in 9 studies.^7^^,^^10^^,^^24^^,^^25^^,^^28^^,^^32–34^^,^^46^ The blinding of outcome assessment was not clearly reported in 7 studies,^7^^,^^10^^,^^24^^,^^28^^,^^33^^,^^34^^,^^43^ with 1 study having high risk of bias.^26^

### Impact of TRF on healthy individuals

Twelve studies investigated TRF supplementation, at dosages of 150 mg to 400 mg daily for periods ranging from 7 weeks to 6 months, in healthy individuals.^4^^,^^5^^,^^9^^,^^10^^,^^24–26^^,^^31^^,^^33–36^ Out of the 12 studies, 2 studies investigated the incidence of PIH and pre-eclampsia in healthy pregnant women,^35^^,^^36^ 2 studies investigated antioxidant and aging parameters,^4^^,^^5^ 4 studies investigated lipid profiles,^24^^,^^32–34^ and 4 studies investigated TRF supplementation on immune parameters.^9^^,^^10^^,^^26^^,^^31^ Significant improvements were observed in 10 studies, of which 3 reported on immunological response,^9^^,^^10^^,^^26^ 3 on cardiometabolic markers^24^^,^^33^^,^^34^ and 4 on oxidative stress.^4^^,^^5^^,^^25^^,^^32^ There were studies that reported significant changes in the placebo group at the end of the trial when compared with baseline in the same group. Azman et al^5^ reported a decrease in catalase activity in their placebo group. Goon et al^4^ reported an increase in malonaldehyde in their placebo group. Another study by Chin et al^32^ reported a significant reduction in glutathione peroxidase (GPX) levels and catalase activity in their placebo group.

### Impact of TRF on pre-existing morbidities

Eighteen studies looked at patients with underlying medical conditions. Significant improvements were reported in 17 studies, supplementing TRF at doses of 100 to 400 mg for a period of 6 weeks to 2 years. Of the 16 studies, 1 reported on postoperative atrial fibrillation,^47^ 7 on type 2 diabetes,^12^^,^^13^^,^^38–42^ and 8 reported on cardiometabolic markers.^7^^,^^8^^,^^27^^,^^28^^,^^35^^,^^44–46^

### Impact of TRF on type 2 diabetes and its complications

Seven studies investigated the effect of TRF on type 2 diabetes and its related complications. In 1 study, conducted by Stonehouse et al,^37^ 90 individuals were supplemented with 420 mg TRF daily for 8 weeks, but no significant improvement was found in any of the type 2 diabetes markers tested. However, another study by Tan et al,^38^ involving patients with diabetic nephropathy, found that daily supplementation of 400 mg of TRF for 8 weeks resulted in a significant decrease in serum creatinine levels. Two additional studies from the same group and participants also reported significant improvements in kidney function when individuals were supplemented with 400 mg of TRF daily for 12 weeks^13^ and 1 year,^12^ including an increase in estimated glomerular filtration rate (eGFR) and urea levels.

In a study conducted by Ng et al^39^ it was observed that supplementing with 400 mg of TRF for 8 weeks resulted in significant improvements in diabetic peripheral neuropathy. The improvements were seen in measures of conduction velocity (for median, sural, and tibial sensory nerves), peak velocity (for median and sural nerves), and peak-to-peak (PP) amplitude (for median nerves). Additionally, the study also found a significant increase in nerve growth factor (NGF) and total cholesterol (TC) levels. Similarly, Chuar et al^40^ found that daily supplementation of 400 mg TRF positively improved conduction velocity (median, sural, and tibial sensory nerves), peak velocity (median and sural sensory nerves), and PP amplitude (sural sensory nerves). In another study conducted by the same group on diabetic retinopathy, it was found that a daily intake of 400 mg of TRF for 8 weeks led to significant reductions in aspartate aminotransferase (AST), alanine transaminase (ALT), and retinal hemorrhage in the right eye of diabetic patients.^40^ The Vitamin E in Neuroprotection Study (VENUS) conducted by Hor et al^42^ found that 400 mg of TRF after 1 year did not improve neuropathic symptoms in diabetic peripheral neuropathy, but significant improvements were observed in lancinating pain. Seven studies did not report any significant reduction in glycated hemoglobin (HbA1c).^12^^,^^13^^,^^37–40^^,^^42^

### Other morbidities

A study conducted by Tan et al^48^ investigated the effect of TRF on children aged 6–12 years with ADHD. The study found that supplementing with 200 mg of TRF daily for 6 months resulted in a nonsignificant improvement in National Institute for Children's Health Quality (NICHQ) Vanderbilt Attention Deficit/Hyperactivity Disorder (ADHD) Teacher Rating Scale (VATRS) scores.

### Impact of TRF on health biomarkers

#### Impact of TRF on oxidative stress

Several studies investigated the effect of TRF supplementation on oxidative stress in healthy individuals. It was observed that TRF supplementation at a dosage of 150 mg per day for a duration of 6 months significantly increased plasma levels of superoxide dismutase (SOD),^5^ decreased plasma levels of glutathione disulfide (GSSG),^5^ malondialdehyde (MDA), and DNA damage.^4^ Furthermore, studies conducted by Chin et al in 2008^25^ and 2011^32^ showed that supplementation with 160 mg of TRF for 6 months resulted in a significant decrease in DNA damage, sister chromatid exchange (SCE) frequency, 8-hydroxyguanosine (8-OHdG) levels, carbonyl content, and advanced glycosylation products, and a significant increase in GPX. Overall, these findings suggest that TRF supplementation may improve biomarkers related to aging in healthy individuals.

#### Impact of TRF on immunological response

Four studies explored the impact of TRF consumption on immune response in healthy individuals. Radhakrishnan et al^31^ found no notable changes in interleukin (IL) 4 (IL-4), interferon-gamma (IFN-γ), T-helper (Th) cell, cytotoxic T-lymphocyte (CTL) cell, B-lymphocyte, and natural killer (NK) cell response in healthy individuals after supplementing with 200 mg of TRF for 8 weeks in the absence of an immunological challenge. However, when an immunological challenge such as tetanus toxoid vaccine (TTV) was administered in healthy individuals supplemented with 400 mg TRF, a significant increase in various immune parameters, such as plasma levels of antibodies to the TTV and IFN-γ and IL-4 and IL-6, was observed after 8 weeks of supplementation.^9^ In another study, there were significant changes in various genes related to immune response and cell signaling with 150 mg of TRF daily for 6 months.^26^ In addition, supplementing 200 mg TRF daily to healthy smokers led to a significant increase in the proliferation of CD4^+^ T lymphocytes and B lymphocytes as well as the CD4^+^ to CD8^+^ ratio after 24 weeks.^10^

#### Impact of TRF on lipid profile

Supplementing healthy individuals daily with 160 mg TRF for 6 months resulted in significant improvements in high-density-lipoprotein cholesterol (HDL-C) and HDL–C/total cholesterol (HDL-C/TC) ratio in individuals who were over 35 years old.^32^ It was also reported in the same study that a significant reduction was observed in HDL-C, HDL-C/TC ratio, and triglyceride levels in adults who were older than 50 years in the placebo group.^32^

In another study, daily supplementation of 160 mg TRF for 8 weeks caused a significant reduction in aortic systolic blood pressure, and a reduction in Augmentation Index (AI).^33^ The same authors reported that lower doses of TRF also had significant health effects. Reductions in pulse wave velocity were observed with 100 mg and 150 mg TRF, and a reduction in AI was seen with 50 mg, 100 mg, and 150 mg TRF.^24^ Supplementation of 200 mg TRF daily for 7 weeks was reported to result in a significant increase in TC and low-density-lipoprotein cholesterol (LDL-C).^34^

There were studies that investigated the effects of TRF in individuals who have some risk factors for cardiometabolic diseases. For example, Heng et al^7^ found that administering 400 mg of TRF daily for 16 weeks resulted in a significant decrease in TC, LDL-C, HDL-C, IL-6, and tumor necrosis factor-alpha (TNF-α) in patients with metabolic syndrome. The same authors also reported a significant increase in TC/HDL-C ratio in the same group of patients.^7^ Another study by Gopalan et al^8^ found that daily supplementation of 200 mg TRF for 2 years led to a significant increase in mean white matter lesions in patients at risk of CVD, but no significant changes were observed in their lipid profiles. Meanwhile, Daud et al^27^ found that administering 180 mg of TRF daily for 16 weeks to individuals with end-stage renal disease led to a significant increase in apolipoprotein A1 (ApoA1) and HDL-C, as well as a significant decrease in plasma triacylglycerol (TG), LDL-C, and cholesteryl-ester transfer. Another study reported a significant improvement in TC and LDL-C levels after supplementing 300 mg of TRF for 6 months to patients with hypercholesterolemia.^46^ However, in another study by Gan et al^43^ investigating the effect of 200 mg TRF daily for 2 weeks, there was no significant improvement in platelet and thrombotic markers in patients with metabolic syndrome.

## DISCUSSION

Palm oil consumption has been on the rise globally. Recent studies have shown that moderate intake of palm oil within a healthy diet presents no risks to health.^49^ Palm oil has been granted a Generally Recognized As Safe (GRAS) status by the US Food and Drug Administration.^50^ Furthermore, the consumption of TRF from palm oil has been found to have numerous beneficial health effects for healthy individuals and those with comorbidities, as observed in this review. In a recent development, palm TRF, a form of vitamin E derived from palm oil with a high tocotrienol content, has been officially recognized as an additional nutrient permitted for use in food products under the Malaysian Food Act of 1983.^51^

Current evidence presented in this systematic review of RCTs strongly supports the consumption of TRF from palm oil as a means of improving various health outcomes among healthy individuals. This includes improvements in immunological response, oxidative stress levels, and lipid profiles. Specifically, daily consumption of TRF at doses ranging from 150 to 400 mg over a period of 8 to 24 weeks resulted in significant improvements in immunological response.^9^^,^^10^^,^^26^ However, 1 study did not find any improvement in the immunological biomarkers as the population was not subjected to any form of exposure to a foreign antigen; hence, no significant changes could be observed.^31^ This is a crucial finding, as a strong immune system is essential for overall health and well-being.

Studies in this review also showed that TRF supplementation can improve various health outcomes in patients with certain health conditions, of which many are associated with chronic inflammation. A number of studies evaluated in this systematic review reported the downregulation of proinflammatory cytokines and lipid peroxidation products, which supports the role of TRF as a potent antioxidant and anti-inflammatory agent. In terms of antioxidant activities, daily consumption of 150–160 mg of TRF over 6 months reduced levels of enzymes related to oxidative stress,^4^^,^^32^ which is known to be a contributing factor to many chronic diseases. However, mixed results were observed when TRF was supplemented to patients who had varying forms of morbidities. Three studies reported no significant improvement when TRF doses ranging from 180 to 400 mg were supplemented for 4 weeks to 6 months.^37^^,^^43^^,^^47^

There is evidence showing the associations between diabetes mellitus and neurodegeneration, ^52^ which share common pathophysiological events, including inflammation and oxidative stress.^53^ Furthermore, a separate systematic review investigated the role of T3 in neuroprotection in preclinical studies and found that TRF and α-T3 exerted antioxidant, antiapoptotic, and anti-inflammatory activities in animal models of neurodegenerative disorders.^54^ Upregulation of the antioxidant systems was also reported in patients receiving TRF.^4^^,^^5^ Persistent and prolonged hyperglycemia tends to lead to severe oxidative stress, which is proinflammatory and associated with nephropathy, neuropathy, and retinopathy. Diabetic neuropathy is a type of nerve damage that can occur due to chronic hyperglycemia. An increase in oxidative stress, activated inflammatory pathways, and a reduction in NGF are some of the general causes of diabetic neuropathy.^39^ A daily dose of 400 mg TRF for 12 weeks to 1 year was found to significantly improve diabetic peripheral neuropathy (improved conduction velocity, peak velocity, PP amplitude, and NGF).^39^ However, 1 study did not report any significant improvement in diabetic neuropathic symptoms following 400 mg of TRF supplementation for 1 year.^42^

Diabetic retinopathy is a common microvascular complication of diabetes, and a 400-mg dose of TRF for 8 weeks was found to significantly reduce diabetic retinopathy (decrease in retinal hemorrhage).^41^ TRF consumption at a dose of 400 mg daily for 12 weeks to 1 year has been shown to improve complications related to nephropathy (reduction in creatinine and increase in eGFR).^12^^,^^13^^,^^38^ Although the studies in our review did not show a reduction in HbA1c, a recent meta-analysis has provided evidence that supplementation of 250–400 mg TRF can significantly reduce HbA1c levels.^55^

A study also points to TRF’s ability to improve insulin resistance in a diabetic animal model via the insulin receptor substrate 1 (IRS-1) and AMP-activated protein kinase/ Sirtuin 1/ Peroxisome proliferator-activated receptor-γ coactivator 1-α (AMPK/SIRT1/PGC1α) pathways. Insulin resistance plays a significant role in muscle atrophy in diabetes mellitus, causing abnormal insulin signaling by reducing tyrosine phosphorylation of the insulin receptor and phosphorylation of Akt. This ultimately affects glucose transport. Supplementation of TRF to diabetic mice upregulated the expression of IRS-1, Akt, and translocation of GLUT-4. Another pathway that was found to be involved in the translocation of GLUT-4 was the AMPK/SIRT1/PGC1α. Supplementation of TRF activated this pathway, leading to improved mitochondrial content and enhanced GLUT-4 expression. The activation of these pathways by TRF improved glucose metabolism and glucose uptake.^56^

Studies that evaluated lipid profiles noted promising changes in various parameters, including TG, HDL-C, LDL-C, TC, and ApoA1. Magosso et al^44^ showed a hepatoprotective effect with 200 mg of TRF daily for 1 year, following which α-T3 was shown to be preferentially distributed in the liver and exerted more potent antioxidant activity in microsomal lipid peroxidation, hence reducing the progression of fatty liver disease. However, 1 study reported no improvement in lipid biomarkers after TRF treatment.^37^ Further analysis revealed that this study had mixed patient populations, which could vary the effect as some patients were found to have varying morbidities (some being diabetic while others were not) in the anthropometric data.^37^ Despite mixed results, the potential benefits of TRF in combating metabolic diseases cannot be overlooked and further research is needed to fully explore its potential. An increase in LDL-C promotes the accumulation of cholesterol within macrophages and other immune cells, which promotes proinflammatory responses,^57^ while an increase in HDL-C counteracts this process by reducing the build-up of inflammation.^57^ However, numerous studies observed no changes in HDL-C^8^^,^^34^^,^^37^^,^^44–46^ and 1 study reported a significant reduction in HDL-C.^7^ Although many studies reported in this review did not show significance, a separate meta-analysis has shown that supplementation of T3 significantly increased the levels of HDL-C.^58^ No changes in HDL-C levels could be due to the long duration of TRF intake (>6 months), resulting in normalized metabolic levels,^8^^,^^44–46^ but does not explain the other 2 studies that had shorter intervention durations of 7 weeks^34^ and 8 weeks.^37^ However, Heng et al^7^ reported a discrepancy in HDL-C reduction that cannot be explained, as previous studies from the same group reported a significant increase in HDL-C.^32^ Natural antioxidants such as TRF hold promise in reducing metabolic risk factors. Specifically, TRF^59^ and δ-T3^60^ isomers have been found to reduce inflammation by lowering cholesterol production in multiple pathways.

The lipid-lowering properties (particularly TG) of T3 were observed on HepG 2 cells and have been attributed to the downregulation of fatty acid synthase (*FASN*) and the upregulation of *CPT1A* and *CYP3A4*. *FASN* is involved in the synthesis of long-chain fatty acids, while *CPT1A* and *CYP3A4* are involved in β-oxidation. Hence, the regulation of these genes partly may explain the TG-lowering effect following T3 intake.^61^ Another in vitro study pointed out that the lipid-lowering properties of T3 may be related to its ability to reduce the accumulation of lipid droplets in 3T3-L1 preadipocytes. These lipid droplets function to store and hydrolyze neutral lipids such as TG and cholesterol esters. Hence, a reduction in these droplets may explain a reduction in TG.^62^

TRF antioxidants have been shown to function as immune-regulating agents, reducing the risk of oxidative stress when the immune system is active.^9^ One bioactivity of T3 is to exhibit potent anti-inflammatory properties and improve immune parameters. Although it is a well-known potent immune enhancer, much of the literature has been focused on tocopherol bioactivity instead of T3. When healthy individuals supplemented with TRF for 56 days were challenged with a booster vaccine (TTV) on day 28, there was a significant increase in the immune parameters measured, such as the level of antigen-specific antibodies (anti-TT IgG), and the production of cytokines, such as IFN-γ and IL-4, with a reduction in IL-6.^9^ However, another study by the same group did not find significant changes in immunological biomarkers in the absence of an immunogenic challenge with the same dosage of 400 mg TRF supplementation in healthy volunteers.^31^ Another study investigated the effect of smoking, which is antigenic and contains free radicals that can adversely affect the immune system.^10^^,^^26^ Supplementation of 150 mg of TRF daily for 6 months was found to significantly increase the proliferation of lymphocytes, CD4^+^ cells, and B cells in the smoking group.^10^ Based on the available evidence, TRF appears to play a role in modulating the immune response.

The role of TRF in immunological response stems from its antioxidant ability to modulate the production of cytokines improving the response of T cells to an antigen. Daily supplementation of TRF enhanced immune response to TTV in BALB/c mice, where the researchers reported that anti-TT IgG production was augmented and there was an increased production of IFN-γ and IL-4, with a significant reduction in TNF-α.^63^ Proinflammatory cytokines such as TNF-α^7^ and IL-6^7,9^ have been shown to be reduced following supplementation of TRF. This could be attributed to γ-T3 suppressing nuclear factor-κB (NF-κB), lowering the secretion of TNF-α and IL-6.^59^^,^^64^ On the other hand, IL-4 was significantly upregulated after supplementation of TRF.^9^ IL-4 plays a role in differentiated T cells to Th2 cells, whereas IFN-γ is involved in the Th1 response.^65^ In addition to its known effects on immune modulation, it is noteworthy to consider the potential of T3 to modulate crucial inflammatory pathways, such as NF-κB,^66^ cyclooxygenase-2 (COX-2),^66^ and signal transducer and activator of transcription factor 3 (STAT3).^67^

Pre-eclampsia is a common disease during pregnancy, which often leads to adverse fetal and maternal events, and a meta-analysis found that different prophylactic strategies, such as aspirin, calcium, vitamin D supplementation, or low-molecular-weight heparin (LMWH), were efficacious in reducing the incidence of pre-eclampsia and PIH.^68^ A study with TRF found that palm tocotrienols significantly reduced the risk of PIH or pre-eclampsia development compared with the control group.^35^^,^^36^ However, this is the only known study to date with TRF and requires further investigation. As the pathophysiology of pre-eclampsia involves both oxidative stress and inflammation activation, TRF, which possesses anti-inflammatory and antioxidant characteristics, can reduce the risk of complications that are associated with pre-eclampsia, such as inhibition of TNF-α–induced activation of NF-κB, regulation of blood pressure, and protection against ischemic injury.^69^

Generally, the majority of the studies reported positive improvements in health-related biomarkers when patients were supplemented with doses ranging from 100 mg to 400 mg TRF daily over 2 weeks to 2 years, but the exact mechanisms through which T3s exert their beneficial properties are not yet fully understood. However, several mechanisms of action have been suggested based on studies conducted in experimental animal models^70^^,^^71^ and cells.^72^^,^^73^ Many of these pathways indicate that TRF supplementation reduces the impact of inflammation on the body's physiological system. The importance of antioxidants in combating oxidative stress and inflammation cannot be overstated, especially in the context of aging and the development of chronic diseases at the cellular level. It is clear from numerous studies that T3 possesses superior antioxidant capabilities that can significantly reduce the accumulation of harmful reactive oxygen species (ROS) and other free radicals.^72^^,^^73^ The key mechanism by which T3 exerts its antioxidant effects is through the stabilization of ROS and other free radicals by donating a hydrogen ion. γT3 has been found to reduce DNA damage by protecting against telomere shortening and increased telomerase activity in human diploid cells.^74^ Moreover, γT3 has been shown to regulate signaling pathways that mitigate radiation damage to DNA, thus providing protection against harmful environmental factors. One study has shown that purified δT3 from palm oil has been shown to reduce intracellular ROS levels and increase the defense systems with an increase in glutathione (GSH)/glutathione disulfide (GSSG) ratio, preventing oxidative damage while modulating the Phosphoinositide 3-kinases (PI3K)/Akt pathway and nuclear factor erythroid 2–related factor 2 (Akt-Nrf2) signaling pathways in MC3T3-E1 cells.^73^

A decrease in MDA, a final product of polyunsaturated fatty acid peroxidation in cells, is also essential, and γT3 has been found to play a role in preventing the reduction of important enzymes such as glutathione peroxidase and superoxide dismutase that are involved in the MDA pathway.^75^ Additionally, a recent meta-analysis suggested that daily consumption of 400 mg of T3 can significantly reduce the production of MDA.^76^ However, it is important to note that this meta-analysis included studies reporting single and/or multiple isomers of T3 supplementation, making it difficult to draw a strong conclusion. Nevertheless, the evidence supports the antioxidant capabilities of T3, and its potential to prevent lipid peroxidation and oxidative stress. However, it is important to acknowledge that these pathways have not been extensively studied in clinical trials, indicating the need for further investigation to fully understand the impactful role of T3 in these mechanisms.

This study has several limitations that should be taken into consideration. First, the review encompassed a wide range of heterogeneous studies, including various diseases instead of focusing on a single disease. In addition, the TRF doses used were in a wide range (50–400 mg daily) for several months and it should be noted that, at present, there is no Recommended Daily Allowance (RDA) for TRF or tocotrienols, which would be useful in making a stronger conclusion in this paper. There is also no uniformity in the duration of the TRF supplementation. Furthermore, this systematic review lacks information to carry out analysis that could determine if TRF is dose dependent, as a majority of the studies that investigated TRF looked at doses that were equal to or more than 400 mg or doses that were equal to or less than 200 mg, as seen in Figure 2.

However, it is important to note that these diseases share a common link with oxidative stress and inflammation. Additionally, the utilization of different formulations of TRF by various commercial companies introduces another source of variation. Since TRF is a commercial product, each formulation may yield different results. Another limitation is the variation in dosages among the studies. Different doses of TRF, ranging from 50 mg to 420 mg, were utilized, which hindered the ability to determine a definitive therapeutic dose for the medical conditions discussed in this review. Furthermore, it is worth mentioning that the majority of the conducted studies were primarily carried out in Malaysia. This is due to the fact that the TRF investigated in the studies is derived from palm oil, and Malaysia is one of the largest producers of palm oil, contributing to a potential regional bias. Furthermore, 3 studies did not report plausible reasons for the significant changes they observed in their placebo group.^4^^,^^5^^,^^25^

It is important to highlight that there is a limited number of clinical studies that have investigated the effects of TRF on immune regulation, despite the well-established recognition of vitamin E as an immune enhancer. Instead, the mechanisms of TRF action on the immune system have been extensively explored using in vitro and in vivo approaches, particularly regarding immune responses and potential anticancer properties.^72^ Finally, due to the heterogeneous nature of the studies examining different biomarkers, a comprehensive meta-analysis could not be conducted to provide a collective analysis of the available data. This scarcity of clinical evidence and the need for further research underscore the importance of future studies to elucidate the precise impact of TRF on immune regulation.

## CONCLUSION

The evidence presented in this review establishes TRF as a valuable nutrient with potential to improve conditions associated with lipid peroxidation, inflammation, and oxidative stress. Despite the varying dosages of TRF, ranging from 50 mg to 420 mg, the current literature consistently demonstrates significant beneficial effects in protecting against oxidation, inflammation, and lipid peroxidation. The multifaceted role of TRF as a therapeutic and prophylactic agent can potentially ameliorate existing conditions or prevent their onset in both healthy individuals and those with morbidities. However, further research must be conducted in areas where TRF shows promise but with limited evidence and studies that remain inconclusive. These include pregnancies characterized by pre-eclampsia and PIH, as well as areas with conflicting results, such as those in lipid studies, which necessitate more robust investigations and clinical trials.

## Supplementary Material

nuae061_Supplementary_Data
