# Supplementary material for: Health Benefits of Palm Tocotrienol-Rich Fraction: A Systematic Review of Randomized Controlled Trials
Source: Nutr Rev. 2024 Jun 25;83(2):307–28. doi: 10.1093/nutrit/nuae061 (PMC11723135; doi:10.1093/nutrit/nuae061)
Supplement: nuae061_Supplementary_Data [file nuae061_supplementary_data.zip › nuae061_Supplementary_Data/Minor revision (Nutrition reviews).docx]

| Comments | Corrections | Rebuttal |
| --- | --- | --- |
| Reviewer 1 | | |
| The last sentence in the Introduction (line 60-62) is a rather premature statement in terms of its location in the manuscript and should be omitted ("The findings from this study will undoubtedly offer valuable insights and information, supporting the use of palm TRF supplementation as an effective therapeutic intervention.") | The authors agree with this statement and has chosen to omit the sentence in the introduction. A similar statement has been written in the conclusion in this systematic review (line 480-483) |  |
| The sentence "Although not significant, a meta-analysis have shown that supplementation of T3 significantly increased the levels of HDL-C" (line 373) seems to contain two contradictory phrases.  I suggest the author look into this and consider amending the sentence. | The authors have rewritten the sentence for further clarity (line 357-359) |  |
| The subtopics "Impact of TRF on Type II Diabetes and its Complications" (line 252) and "Other morbidities" (line 279) should come under the subtopic "Impact of TRF on pre-existing morbidities" (line 191). | The authors has agreed to move the subtopics to a more appriopriate section |  |
| In discussing the role of TRF in preeclampsia and PIH (lines 419-425), I suggest the author refer to a related publication (Mahdy, Z.A.; Chin, K.-Y.; Nik-Ahmad-Zuky, N.L.; Kalok, A.; Abdul Rahman, R. Tocotrienol in Pre-Eclampsia Prevention: A Mechanistic Analysis in Relation to the Pathophysiological Framework. Cells 2022, 11, 614. https://doi.org/10.3390/cells11040614). There is also a typographical error in line 420 - "material" should be replaced by "maternal | Authors have further discussed the role of TRF in pre-clampsia (line 410-414) and replaced the typographical error at line 405. |  |
| Lines 497-527 should be placed under Discussion, not Conclusion. | The authors has moved the limitations to the discussion section |  |
| The phrase "unequivocally establishes" should be omitted from the Conclusion to avoid bias (line 492).  The Conclusion should also be expanded to include the words "therapeutic" and "prophylactic" - the two roles of TRF - echoing the objective of the review as stated in the Introduction.  The Conclusion should also mention the need for further exploration and research into certain areas where there seems to be potential benefit yet evidence is still lacking, e.g. in pregnancies at high risk of preeclampsia and PIH, and areas where evidence is contradictory such as in the lipid studies, etc. | The authors agree that the phrase "unequivocally establishes" is a bias remark and has agreed to remove the phrase. The conclusion has been expanded as per suggestion of the reviewer (line 480-485) |  |
| Reviewer 2 | | |
| Authors have addressed all the comments accordingly. This manuscript can now be accepted for publication by Nutrition Reviews | The authors would like to thank reviewer two for the recommendation |  |
| Editors | | |
| - The PICOS criteria used to define the research question are presented as Table 1 and the table is cited at an appropriate place in the text. | Authors have added table 1 which includes the inclusion and exclusion criteria for this study |  |
